# Nociplastic pain, central sensitization, and mixed pain: a critical reflection from clinical practice and evidence

**DOI:** 10.3389/fmed.2026.1815975

**Published:** 2026-04-01

**Authors:** Antonio Alcántara Montero

**Affiliations:** Centro de Salud Trujillo, Trujillo, Cáceres, Spain

**Keywords:** central sensitization, chronic pain mechanisms, mixed pain, nociplastic pain, pain phenotyping, primary care

Chronic pain has undergone a profound conceptual transformation in recent years, yet this shift remains only partially reflected in everyday clinical practice. Globally, chronic pain affects nearly one in five adults (1), and Primary Care absorbs the majority of these consultations, making mechanistic clarity not an academic luxury but a clinical necessity. For decades, clinicians relied on a binary nociceptive–neuropathic classification that attempted to impose order on the complexity of human pain. While historically useful, this dichotomy is insufficient to explain the reality of many patients seen in Primary Care: individuals with persistent pain, marked disability, and a striking mismatch between symptom severity and structural findings. Recent literature echoes this impression and calls for a more integrated understanding of chronic pain mechanisms (2).

This Opinion Article argues that nociplastic pain, central sensitization, and mixed pain should be integrated into a unified, clinically actionable framework for Primary Care, where most chronic pain is managed under conditions of limited diagnostic resources and time constraints. A pragmatic, phenotype-oriented approach is proposed, repositioning central sensitization as a primary therapeutic target and recognizing mixed pain as the prevailing clinical presentation rather than an exception. This perspective aims to offer a more realistic and useful interpretation of pain mechanisms in the clinical setting where simple, applicable tools are most needed.

The introduction of the mechanistic descriptor “nociplastic pain” in 2017 attempted to fill a long-standing conceptual gap. Defining pain arising from altered nociception without clear evidence of tissue damage or somatosensory system injury provided a framework for patients who had long been confined to ambiguous labels such as “nonspecific pain.” The 2021 clinical criteria represented an important step forward by offering a structured approach based on pain duration, distribution, mechanistic plausibility, evoked hypersensitivity, and associated comorbidities (3). These criteria have been contextualized within precision medicine and the broader theoretical basis of central sensitization (4, 5), and recent reviews have highlighted both their usefulness and their limitations (6).

Despite their value, the criteria present important challenges. Evoked hypersensitivity is not exclusive to nociplastic pain; regional or multifocal distribution can appear in inflammatory or neuropathic conditions; and associated comorbidities lack specificity. Several authors have warned of the risk of overdiagnosis and the drift of nociplastic pain into a conceptual “catch-all” category (6). These limitations underscore the need to interpret nociplastic features not as a discrete diagnostic entity but as part of a mechanistic continuum in which central sensitization plays a transversal role.

Central sensitization—understood as an amplification of nociceptive processing within the central nervous system—appears across a wide range of musculoskeletal, rheumatologic, and visceral conditions (4). Findings such as generalized hyperalgesia, allodynia, enhanced temporal summation, and impaired conditioned pain modulation consistently emerge in laboratory and clinical research. However, in Primary Care, central sensitization and nociplastic pain remain underrecognized, despite their growing relevance for everyday clinical decisión-making (7, 8). This underrecognition is not merely conceptual; it has practical consequences. When clinicians lack a mechanistic framework, they tend to default to structural explanations, escalating imaging, referrals, or peripherally focused treatments that may not align with the underlying biology of the patient's pain.

Nociplastic pain is frequently embedded within broader musculoskeletal phenotypes. For example, knee osteoarthritis often presents with mixed nociceptive and nociplastic features, and central sensitization significantly alters prognosis and treatment response (9). This reinforces the need to move beyond structural interpretations of pain and toward mechanism-based reasoning. The same applies to chronic low back pain, shoulder pain, and widespread musculoskeletal pain, where nociplastic features often coexist with nociceptive drivers, creating a complex clinical picture that cannot be adequately addressed by unimodal interventions. A typical scenario in Primary Care is the patient with chronic low back pain, mild degenerative findings on imaging, neuropathic descriptors, and widespread tenderness—an everyday example of how mixed mechanisms converge in a single clinical presentation.

Recent systematic evidence shows that central sensitization and neuropathic—like pain features are associated with poorer outcomes and increased risk of persistent pain following peripherally focused interventions (10). This finding has profound implications for Primary Care, where decisions about referrals, imaging, and procedural interventions are often made early in the clinical course. Recognizing sensitization features can help clinicians avoid unnecessary or ineffective interventions and redirect patients toward multimodal strategies with greater potential for benefit.

In parallel, the concept of mixed pain has emerged to describe the coexistence of nociceptive, neuropathic, and nociplastic mechanisms within the same patient. Evidence shows that this overlap is not anecdotal but extremely common in conditions such as chronic low back pain with radiculopathy, osteoarthritis with sensitization, cancer pain, persistent postsurgical pain, and chronic musculoskeletal syndromes. Mixed pain is increasingly recognized as a distinct clinical challenge in Primary Care (11). Recent international recommendations emphasize that mixed pain is highly prevalent, clinically burdensome, and frequently underdiagnosed, and that its management requires structured, mechanism-based and multimodal strategies (12). Attempting to fit these patients into a single mechanistic category is not only unhelpful but clinically detrimental. Mixed pain should therefore be understood not as a residual category but as the predominant clinical pattern in Primary Care (13, 14).

A recent international consensus on low back pain phenotyping offers a structured approach based on duration, distribution, mechanistic plausibility, evoked hypersensitivity, historical hypersensitivity, and comorbidities (15). Although developed for a specific condition, this model provides a conceptual foundation for broader mechanistic reasoning. Building on this evidence, a minimum viable clinical screening approach for Primary Care can guide decision-making within brief consultations.

This approach can be organized around three key clinical questions: (I) Is there a mismatch between symptom severity and structural findings? (II) Are there neuropathic descriptors or signs suggesting neural involvement? (III) Are there features of hypersensitivity or comorbidities consistent with central sensitization?

These questions can be complemented by three clinical signs: evoked hypersensitivity (mechanical or thermal), generalized hyperalgesia or widespread tenderness, and pain disproportionate to movement or load. Together, they lead to three therapeutic implications: prioritizing central modulation strategies when sensitization features are present; combining modalities in mixed pain; and avoiding overreliance on peripherally focused interventions when nociplastic features predominate. This minimum viable screening framework is conceptual and derived from expert clinical reasoning. It has not yet undergone empirical validation and should therefore be interpreted as a pragmatic heuristic rather than an evidence-based diagnostic tool.

The realities of Primary Care add another layer of complexity. Limited time, scarce diagnostic tools, and high workload pressures favor rapid decisions based on simplified models. As a result, many patients with nociplastic or mixed pain receive treatments focused on peripheral tissues that do not address the underlying mechanisms and may reinforce inaccurate beliefs about structural damage. The lack of specific training in central sensitization and nociplasticity exacerbates this problem (8). Fragmented healthcare systems further hinder access to coordinated multimodal interventions. Moreover, the cultural expectation—shared by clinicians and patients—that pain must have a visible structural cause continues to shape clinical encounters, often leading to frustration, unnecessary investigations, and therapeutic inertia.

From a broader perspective, the shift toward mechanistic classification also intersects with health-system priorities. Overuse of imaging, procedural interventions, and specialist referrals contributes to rising healthcare costs without improving outcomes. Mechanism-based reasoning offers an opportunity to realign clinical practice with value-based care by promoting early identification of patients unlikely to benefit from peripherally focused treatments and redirecting them toward multimodal, non-invasive strategies. This is particularly relevant in Primary Care, where clinicians must balance clinical uncertainty with resource stewardship.

From a critical perspective, the greatest risk of the nociplastic pain construct is its potential drift into a category that encompasses everything we do not fully understand. Avoiding this drift requires solid operational criteria, research validating their clinical utility, and training that enables clinicians to apply them rigorously (2, 3, 6). Similarly, mixed pain requires formal recognition and diagnostic tools that allow its components to be identified with greater precision (11–14). Mechanism-based classification should not be a theoretical exercise but a practical guide for therapeutic decision-making.

Understanding pain is not merely about classifying it but about interpreting its complexity to guide more precise and person-centered clinical decisions. Recent literature offers a richer and more nuanced conceptual framework, but real transformation will depend on the ability to translate it into clinical practice. The next step is to develop practical tools, training pathways, and decision frameworks that allow Primary Care clinicians to operationalize mechanistic reasoning in real time. This requires specific training, adequate consultation time, appropriate diagnostic tools, and a cultural shift that allows these concepts to be integrated into Primary Care. Only then will clinicians be able to offer patients an approach that is truly centered on their needs and aligned with the mechanisms driving their pain.
